# Community-oriented integrated care and health promotion – views from the street

**DOI:** 10.1080/17571472.2015.1082347

**Published:** 2015-09-28

**Authors:** Paul Thomas, Tony Burch, Ewan Ferlie, Rachel Jenkins, Fiona Wright, Amrit Sachar, Baljeet Ruprah-Shah

**Affiliations:** ^a^ London Journal of Primary Care, RCGP London, London, UK; ^b^ Health Education NW London, London, UK; ^c^ Dept of Management, King’s College London, London, UK; ^d^ King’s College London; ^e^ Global Health, Queen Mary’s University, London, UK; ^f^ Liaison Psychiatry Services, West London Mental Health NHS Trust; ^g^ Accomplish Consultancy

**Keywords:** Integrated care, community-oriented integrated care, primary care, NHS

## Abstract

On the 1st and 2nd May 2015, participants at the RCGP London City Health Conference debated practical ways to achieve integrated care at community level. In five connected workshops, participants reviewed current work and identified ways to overcome some of the problems that had become apparent. In this paper, we summarise the conclusions of each workshop, and provide an overall comment. There are layers of complexity in community-oriented integrated care that are not apparent at first sight. The difficult thing is not persuading people that it matters, but finding ways to do it that are practical and sustainable. The dynamic and complex nature of the territory is bewildering. The expectation of silo-operating and linear thinking, and the language and models that encourage it, pervade health and social care. Comprehensive integration is possible, but the theory and practice are unfamiliar to many. Images, theories and models are needed to help people from all parts of the system to see big pictures and focused detail at the same time and oscillate between them to envision-integrated whole systems. Infrastructure needs to enable this, with coordination hubs, locality-based multidisciplinary meetings and cycles of inter-organisational improvement to nurture relationships across organisational boundaries.

## Why this matters to us

London Journal of Primary Care has long advocated community-oriented integrated care as a key aspect of an efficient and effective NHS. This was one of the key themes at the May 2015 City Health Conference organised by RCGP London and LJPC. Each of five workshops cascaded ideas to the next to build up a rich understanding of how to make community-oriented integrated care work. Our concern is to subject these ideas to broad critique and explore the best ways to create an efficient and effective NHS.

## Key messages

### Resources

• If 90% of NHS care takes place in primary care, it is not right that primary care share of NHS spend has declined from around 11 to 8.4% in recent years.
• Primary care leadership of integrated care needs investment over and above the funds needed to deliver services.
• Comprehensive integration is possible, but the theory and practice is unfamiliar to many and it requires training for practitioner and manager at all educational levels.

### Principles

• Market approaches tend to fragment rather than integrate services – this tendency might be counteracted if collaboration is mandated from everyone.
• An infrastructure of facilitation and communication is needed to establish high performing teams across organisational boundaries.
• IT systems need to support shared care, shared leadership and self-help.

### Models

• Evaluated case studies of enabling approaches to integrated care, cultural change and community development are needed.
• Relationship-based, empowering practice with inter-organisational learning needs to become the dominant approach throughout the NHS.
• The potential for community hospitals to support community-oriented integrated care needs to be rediscovered.

### Good mental health

• Clusters of general practices need to develop local communities for health that facilitate collaborative learning and coordinated change between different disciplines.
• All agencies need to advocate for good mental health at all stages of life, from preconception to end of life, with well-managed transitions between life stages.
• All citizens and organisations need to engage in collaborative action to promote good mental health – movement, nutrition, positive parenting and community development.

## The City Health Conference 2015

On the 1st and 2nd May 2015, participants at the RCGP London City Health Conference debated practical ways to achieve integrated care at community level. In five connected workshops, each of an hour duration, participants reviewed current work, and identified ways to overcome some of the problems that had become apparent.

In this paper, we summarise the conclusions of each workshop, and provide an overall comment.

The five workshops included presentations of the following content:

(1) Ewan Ferlie on the ideas that he put forward in a LJPC paper [1] in which he critiqued Lord Darzi’s vision for integrated care in London.[2]
(2) Tony Burch on the work done in Brent to use community hospitals as a focus for integrated working between generalist and specialist practitioners.
(3) Rachel Jenkins and Fiona Wright on the work in progress of an expert learning set sponsored by the charity ETHICS, and supported by London Journal of Primary Care.
(4) Amrit Sachar and Paul Thomas on learning from locality workshops of the Integrated Care Programme in West London.
(5) Baljeet Ruprah-Shah on learning from community engagement initiatives in West London Clinical Commissioning Groups.

## Workshop one: four challenges to integrated care need to be considered

Ferlie’s full commentary [1] on Lord Darzi’s vision for integrated care [2] included four challenges. Participants discussed these challenges and agreed that success requires strategy for integration that is shared between local organisations. It needs to combine bottom-up and top-down approaches and to systematically build relationships throughout whole systems of care. To achieve this, unfamiliar theories of *organisational learning* and *complex adaptive systems* are likely to be helpful.

## Challenge one: can primary care development and investment be protected in a cold financial climate?

Primary care leadership of community-oriented integrated care needs investment above the funds needed to deliver services.

Participants argued that 90% of care takes place in primary care, but primary care share of NHS spend has declined from around 11 to 8.4% in recent years [REF MAJEED IN THIS ISSUE OF LJPC]. There is a need to improve the balance of funding between hospitals and primary care, and to fund leadership of community-oriented integrated care. Case studies of collaborative and enabling approaches to cultural change, community development and quality improvement need to be established, and their short- and long-term effects evaluated. Multidisciplinary shared leadership through networks needs to be supported. Good ways to manage conflicts of interest need to be developed.

• Systematic development of primary care in Tower Hamlets was cited as an example of good practice.

## Challenge two: does the current marketisation of health care erode integration?

Participants agreed that market approaches tend to fragment services rather than integrate them. This tendency might be counteracted if the intention to collaborate is written into everyone’s contracts and monitored. An infrastructure of facilitation and communication between different providers of care is needed, to build relationships and teams across organisational boundaries. It is important that all practitioners become skilled at taking a wider, longer-term view to see how their roles fit into the context of the wider health service. ‘Preferred providers’ can be better at facilitating integration when they already have established networks of communication that can be used to build strong teams.

## Challenge three: does the health and social care system behave like a real system?

Participants argued that the language and practice of health care emphasises the ‘silo’ nature of the operation. For example, the term ‘discharge’ implies a sharp distinction between primary and secondary care. It would be better to use the language of ‘shared care’ in which primary and secondary care play complementary roles. Both need to emphasise that the patient is at the centre of their care, and both need to encourage self-management. IT systems need to facilitate shared care, shared leadership and self-help.

• The integrated case management system of Waltham Forest was cited as an example of good practice.

## Challenge four: is there really a cultural shift to empowerment and organisational learning?

Participants agreed that there are not enough examples of bottom-up, enabling approaches to integrated care. It is common to find practices that operate advanced empowerment practice side by side with others that operate a quite different approach. New approaches to cultural change and community development are needed to translate such practice from a few advocates to become the dominant approach throughout healthcare.

There is a need to better understand what happens inside a *complex adaptive system.* This recognises that dynamic interaction between different organisations and disciplines is the way that things naturally evolve – a process of cultural change that builds relationships and gains local ownership. The practical implications of this are poorly understood. Overly frequent staff changes and conflicting demands can damage trusted relationships that are necessary to sustain integration; we should understand better the structures and processes that help to reinvigorate these relationships.

• The reduction of waiting lists in Ealing from joint learning and buddying practices was cited as an example of good practice.
• Relational markets, for example BMW, can develop long-term relationships with its customers.
• There is a need to create a directory of innovations, so those who wish to develop an empowering, organisational learning approach can be informed by other models.
• Strategic thinking across London is needed to systematically develop case studies of organisational learning.
• PhD and other students (e.g. Darzi Fellows) should be encouraged to research case studies of organisational learning.
• There is the need to combine bottom-up innovation with top-down strategy.

## Workshop two: community hospitals could provide an intermediate care hub to support community-oriented integrated care

Participants agreed that integrated care requires a local coordinating function that helps local general practices to work effectively within the whole system. Community hospitals are well placed to provide a coordinating function that could manage ‘step-up’ and ‘step-down’ to and from hospital care. This local function needs to operate in the context of London-wide hospital bed control and borough-wide strategy.

Participants considered two questions:

*What should be the future role of community hospitals?*

Participants noted that Cuba, China and other low-income countries use community hospitals effectively to develop relationships that integrate local services. In the 1980s, England did the same, as does Scotland now. The value of this function needs to be rediscovered. Participants suggested:

• Community hospitals should not duplicate the work of acute hospitals.
• Diagnostics including imaging and other common tests could be done.
• Community services could be co-located, including nurses and rehabilitation teams.
• Health promotion coordination.
• Therapies to maintain mobility and muscle bulk in patients.
• Liaison with secondary care, to help step down, especially for patients who cannot go directly home.
• Rehabilitation and stop-gap care for those who cannot cope at home.
• Admissions avoidance teams.
• Coordination of education and decision support for clinicians.
• Strategic support for networking between professionals.
• Leadership of local improvement projects.

*How should practices and their staff relate to community hospitals and their staff?*

• Liaison that includes in-reach and out-reach.
• Senior medical input, in addition to nurse leadership.
• Generalists need on-going support for medical updating from a range of specialists.
• GPs can work closely with consultant geriatricians, psychogeriatricians and allied health professionals.
• Structured clinical updating, including ward rounds, case studies and learning from multiprofessional assessment clinics.
• Shared community development projects.
• Shared records.
• Form multiprofessional teams around patients with care plans.
• Lead improvement projects, using GP trainees, public health and academics.

## Workshop three: integration of mental health promotion and prevention into primary care

Participants agreed that primary care (in commissioner and provider roles) needs to advocate for mental health at all stages of life, from pre-conception to end of life, with well-managed transitions between different life stages. Often simple and obvious things, repeatedly done, improve mental health – exercise, movement, nutrition, green spaces, social networks and laughter. Positive parenting, relationship-building and community development are important, and schools have a role in nurturing these. Structured approaches to case finding as well as opportunistic interventions using ‘teachable moments’ cleverly are needed. Community involvement and inter-sectoral working are central to addressing mental health. Clinical Commissioning Groups and Health and Well-being Boards have a key role in supporting these.

Participants considered three questions:

*What opportunities exist to integrate mental health promotion and prevention as commissioners and providers?*

• Opportunistic assessment and management by clinicians of anxiety and depression – so-called “teachable moment”.
• Use a sequence of appointments to continue discussions about mental health – i.e. rather than one session.
• Courses for patients on positive mental health, resilience and self-care.
• Practice-based health promotion groups.
• Advertise in waiting rooms programmes to support mental health, e.g. yoga classes, exercise, debt management and post-natal depression.
• Build primary care teams capable of considering mental health at all stages of life.
• Consider mental health when monitoring chronic diseases.
• Consider including questions about stress management in new patient checks and others.
• Targeted interventions at known flash points, like birthday cards, to vulnerable adolescents.
• We can model healthy workforce practices and proactively support our own mental health.
• Clusters of practices have an opportunity to work with local services, e.g. schools, to support local working.
• CCGs and health and well-being boards provide an opportunity to lead inter-sectoral working.

*What are the challenges in integrating mental health promotion and prevention?*

• Insufficient time in appointments; mental health problems can take a long time to explore.
• The people that need the most help usually have many other complaints that compete for attention.
• Problems of children and adolescents tend to present in school and can be disconnected from general practice.
• Perception that social media can encourage self-harm, suicide/eating disorders.
• Long wait for appointments at Child and Adolescent Mental Health Services in some areas.
• Lack of integration between different parts of the NHS.
• Overwhelmed staff.

*What will support us to integrate mental health promotion and prevention into our work?*

• Time, money, more mental health and allied health staff, advice-lines, ‘a friend to phone’.
• Integrated and cross-sectoral approaches to working.
• Sharing information and having access to information.
• Initiatives to reduce stigma.
• Mental health education classes (rather than therapies only).
• Parenting classes – parents are a massive influence on the way children behave and their mental health outcomes.

## Workshop four: multidisciplinary locality meetings can help people to creatively interact, from which develop trusted relationships for longer-term collaboration

Participants agreed that regular creative interaction between different disciplines improves relationships. Locality meetings should be developed to do this, while leading improvement projects and mutual learning. This helps to build teams and communities that in turn enable difficult problems to be solved at low cost. Participants considered two questions:

*What helps locality-based meetings like this to work optimally?*

Participants noted that established meetings could operate at a higher level of sophistication, but they needed periodic stimulation to move to higher levels of operation to avoid becoming stuck in ruts. They operated well when there was:

• Good leadership.
• Multidisciplinary discussions.
• Organisational and administrative support to maintain momentum and keep things running smoothly.
• Data about the success of projects and the overall effect of the initiative.
• Easy communication and advice outside of meetings, including participants contact details.
• Participation of hospital clinicians and link with discharge planning.
• Incentivisation (money, resources, acclaim, continual professional development points).
• Cross-pollination of ideas with other such meetings and a degree of friendly competition between them.

*How should we develop such initiatives in the future?*

Participants noted that there are many initiatives from which to learn that have combined top-down and bottom-up approaches:

• Comprehensive list of contact details – Waltham Forest.
• Political support for multidisciplinary teams – Tower Hamlets.
• Continuing education provider network to support relationship-building – Barnet.
• Evaluated collaborative interventions to reduce admissions – South Bedfordshire.
• Social care unit – Torquay.
• Leadership for integrated care programme – London Leadership Academy.

Participants noted development needs:

• Successful virtual meetings.
• Improved use of electronic communication.
• Leadership skills to design and improve models of operating.
• Combining large group stakeholder meetings with smaller project teams to create networks of networks.

## Workshop five: integrated care requires strong community support to create a culture of participation and collaboration

Participants agreed that practices and clusters of practices need to systematically develop local communities for health. This requires:

• Encouraging champions at all levels.
• Sharing enthusiasm and inspirational vision that will recruit others.
• Look for opportunities to start wherever you can.
• Train users and carers to lead community development.
• Recognition of importance of start-up schemes, including non-recurring funds which are often led by community organisations.
• Having realistic and long-term goals.
• Long-term strategy to facilitate on-going community development.
• Use of local intelligence and surveillance data.

Things that are often forgotten:

• Stand back and develop a visionary future with people from different organisations and backgrounds.
• Create opportunities for people to come together and have fun.
• Need to go out to communities – libraries schools, etc. and not merely expect them to come to practices.
• Reach out to community groups as a cluster of practices – more effective than each practice making individual approaches – and share positive learning.
• We may not know communities as well as we think. Methods such as community diagnosis can help to understand them better.
• Community development is a messy interactive process that goes forwards and backwards. A long-term strategy is essential.
• The boundaries between professionals and communities are often artificial – we are all community members and recognising this will encourage collaborative working.
• Faith groups, schools and voluntary groups are full of people who are developing local communities; integration with their work can be valuable.
• Use statutory bodies to advantage, for example, Health and Well-Being Boards.

Innovative things that others have done:

• Fund practices to visit community groups and write a report for the Clinical Commissioning Group - Wandsworth CCG.
• Formal links with community groups -Tower Hamlets CCG.
• Patient representative on every board - Hackney CCG.
• Users involved in creating agendas for meetings – Healthwatch.
• User panel (patients) and community group members on committees and subgroups – Central London CCG.

## Comment

There are layers of complexity in community-oriented integrated care that are not apparent at first sight. The difficult thing is not persuading people that it matters, but finding ways to do it that are practical and sustainable. The dynamic and complex nature of the territory is bewildering. The expectation of silo-operating and linear thinking, and the language and models that encourage it, pervade health and social care and the training of practitioners, academics and managers. There is barely the language to explain the difference between specialists who need to drill down to specific problems and generalists who need to integrate all aspects of health. There is much to be done, and undone.

Comprehensive integration is possible, but the theory and practice is unfamiliar to many. Images, theories and models are needed to help people from all parts of the system to see big pictures and focused detail at the same time and oscillate between them to envision-integrated whole systems. Infrastructure can enable this with coordination hubs, locality-based multidisciplinary meetings and cycles of inter-organisational improvement to nurture relationships across organisational boundaries.

Mental health care and health promotion need to be considered in all aspects of care. Mental health (as opposed to mental illness) in particular emphasises integration – integration within an individual as a unique identity with their ‘narrative unity’, and as a citizen within vibrant, healthy communities.

Ferlie’s four challenges need to be considered seriously. Integrated care cannot be achieved without investment. Market approaches work against integration. The need to collaborate for the sake of the system as a whole needs to be written into everyone’s contracts. Clinicians and managers need to learn how to design and lead systems that discourage silo-operating and systematically build relationships across organisational boundaries. They need to co-create a culture of empowerment and organisational learning throughout the NHS.

You can continue this discussion. Go to http://www.londonjournalofprimarycare.org.uk/
